# Ovatodiolide triggers ferroptosis in high-grade serous ovarian cancer through HMOX1 upregulation

**DOI:** 10.1016/j.omton.2025.201023

**Published:** 2025-07-21

**Authors:** Yew-Min Tzeng, Aye Aye Khine, Hsuan-Shun Huang, Tang-Yuan Chu

**Affiliations:** 1 Department of Applied Science, National Taitung University, Sec. 2, University Road, Taitung 95092, Taiwan, ROC; 2 Department of Applied Chemistry, Chaoyang University of Technology, Taichung 41349, Taiwan, ROC; 3 Center for Prevention and Therapy of Gynecological Cancers, Department of Research, Hualien Tzu Chi Hospital, Buddhist Tzu Chi Medical Foundation, Hualien 970, Taiwan, ROC; 4 Department of Obstetrics & Gynecology, Hualien Tzu Chi Hospital, Buddhist Tzu Chi Medical Foundation, Hualien 970, Taiwan, ROC; 5 Institute of Medical Sciences, Tzu Chi University, Hualien 970, Taiwan, ROC

**Keywords:** high-grade serous ovarian carcinoma, ovatodiolide, heme oxygenase 1, ferroptosis

## Abstract

High-grade serous ovarian carcinoma (HGSC) is the most aggressive and lethal gynecological malignancy, largely due to its asymptomatic early stages and late-stage diagnosis. Current standard treatments involve surgical resection combined with platinum-based chemotherapy, yet recurrence rates remain high, highlighting the urgent need for more effective therapeutic options. Ovatodiolide, a bioactive macrocyclic diterpenoid derived from traditional medicinal herbs, has been reported to possess anti-cancer properties against various malignancies. In this study, we demonstrated that ovatodiolide exerts potent cytotoxic effects on both HGSC cells and their precursor cells. RNA sequencing (RNA-seq) analysis reveals that the cytotoxicity of ovatodiolide is associated with the upregulation of heme oxygenase 1 (HMOX-1), along with the activation of oxidative stress and ferroptosis, suggesting a distinct cell death mechanism. These findings demonstrate that ovatodiolide induces HGSC cell death through a unique mode of action and highlight its potential as a promising therapeutic agent to complement or enhance existing treatment strategies.

## Graphical abstract

**Figure:**
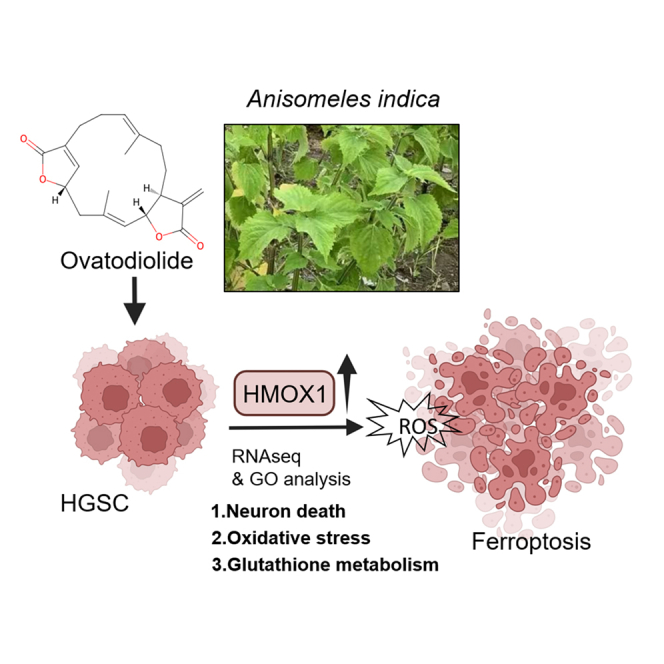

## Teaser

Ovatodiolide, a bioactive macrocyclic diterpenoid derived from the traditional medicinal herb Anisomeles indica, induces potent cytotoxicity in high-grade serous ovarian carcinoma (HGSC) cells by upregulating HMOX1 and activating ferroptosis. This study is the first to reveal its therapeutic potential against HGSC via a ferroptosis-mediated mechanism.

## Introduction

High-grade serous carcinoma (HGSC) is the most common and aggressive type of epithelial ovarian cancer, primarily originating from the epithelium of the fallopian tube fimbriae, with the *in situ* carcinoma lesion referred to as serous tubal intraepithelial carcinoma (STIC).^1^^,^^2^^,^^3^ HGSC is often diagnosed at an advanced stage due to its asymptomatic nature or nonspecific early symptoms, making early detection highly challenging.^4^^,^^5^

Despite efforts to improve early detection have been made, large-scale trials, such as the Prostate, Lung, Colorectal, and Ovarian (PLCO) Cancer Screening trial, have shown no significant reduction in ovarian cancer mortality through routine screening.^4^ HGSC is primarily treated with a combination of surgery and platinum-based chemotherapy, but the frequent resistance to treatment and recurrence underscores the urgent need for the development of novel therapies.^6^^,^^7^^,^^8^

Ovatodiolide, a macrocyclic diterpenoid compound naturally found in Anisomeles indica, is a traditional medicinal herb growing in subtropical regions below 500 m elevation, particularly in Guangzhou and Taiwan, and has historically been used in the traditional medicine of the Hakka people of the Han ethnic group. Recent research has demonstrated its promising anti-cancer properties through multiple mechanisms. Ovatodiolide, in combination with antrocin, synergistically inhibits the stemness and metastasis of hepatocellular carcinoma cells.^9^^,^^10^ It also reduces the oncogenicity of glioblastoma cells by suppressing cancer-stem-cell-like phenotypes.^11^ In oral cancer, it prevents transformation by downregulating exosomal microRNA-catenin cargo,^12^ whereas in endometrial cancer, it inhibits cancer stemness through ROS-mediated DNA damage.^13^ Notably, toxicology studies have demonstrated a favorable safety profile, showing acute oral toxicity exceeding 1,000 mg/kg body weight in rats, with no significant adverse effects at doses ranging from 10 to 50 mg/kg, and no evidence of genotoxicity.^14^ Ovatodiolide emerges as a promising candidate for anti-cancer drug development.

In this study, we utilized STIC-mimicking cells, such as FT282-CCNE1, and the HGSC cells, such as OVSAHO and KUROMACHI, to evaluate the therapeutic potential of ovatodiolide both *in vitro* and *in vivo*. The results demonstrated that ovatodiolide significantly inhibited the *in vitro* growth of these carcinoma cells and, through oral administration *in vivo*, effectively suppressed the growth of OVSAHO-induced orthotopic HGSC ovarian cancer. Cellular RNA sequencing (RNA-seq) analysis further revealed that ovatodiolide exerts cytotoxic effects through mechanisms involving oxidative stress and ferroptosis.

## Results

### Ovatodiolide exhibits dose-dependent cytotoxic effect on both STIC-mimicking cells and HGSC cells

**Figure 1:**
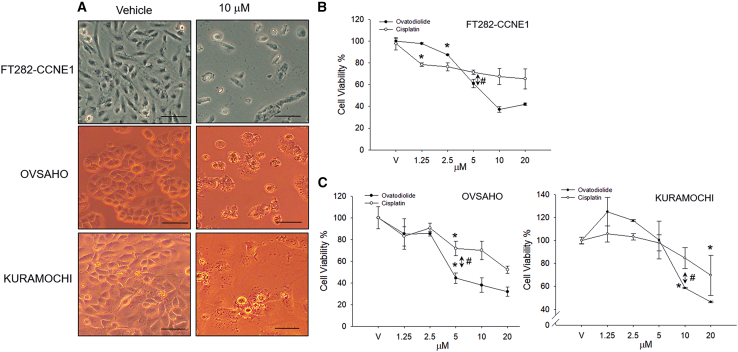
Ovatodiolide demonstrates a dose-dependent cytotoxic effect on both STIC-mimicking cells and HGSC cells (A) Representative images of STIC-mimicking cells (FT282-CCNE1) and HGSC cells (OVSAHO and KURAMOCHI) following treatment with 10 μM ovatodiolide for 48 h. Scale bars, 25 μm. (B and C) Cell viability after 48 h of treatment with varying doses of ovatodiolide or cisplatin. ∗*p* < 0.05 compared to vehicle medium control. #*p* < 0.05 comparison shown in the figure.

To investigate the cytotoxic effect of ovatodiolide on the HGSC cells, we used both STIC-mimicking cells (FT282-CCNE1) and HGSC cells (OVSAHO and KURAMOCHI) for the examination. Figure 1A shows the cell morphological changes 48 h after treating with 10 μM ovatodiolide. Figures 1B and 1C present quantitative data showing that both ovatodiolide and cisplatin exhibit dose-dependent cytotoxic effects on all three cell lines. In FT282-CCNE1 cells, ovatodiolide and cisplatin, exhibited statistically significant cytotoxic effects at concentrations of 2.5 μM and 1.25 μM, respectively (Figure 1B). In contrast, in HGSC cells, significant cytotoxic effects were observed at 5 μM in OVSAHO cells and at 10 μM and 20 μM in KUROMACHI cells for ovatodiolide and cisplatin, respectively (Figure 1C). Notably, ovatodiolide demonstrated superior cytotoxicity compared to cisplatin at concentrations above 5 μM in FT282-CCNE1 and OVSAHO cells and concentrations above 10 μM in KURAMOCHI cells. These data suggest that ovatodiolide effectively inhibits the growth and survival of both STIC-mimicking cells and HGSC cells.

### Ovatodiolide significantly reduces clonogenicity and anchorage-independent growth in STIC-mimicking and HGSC cells

**Figure 2:**
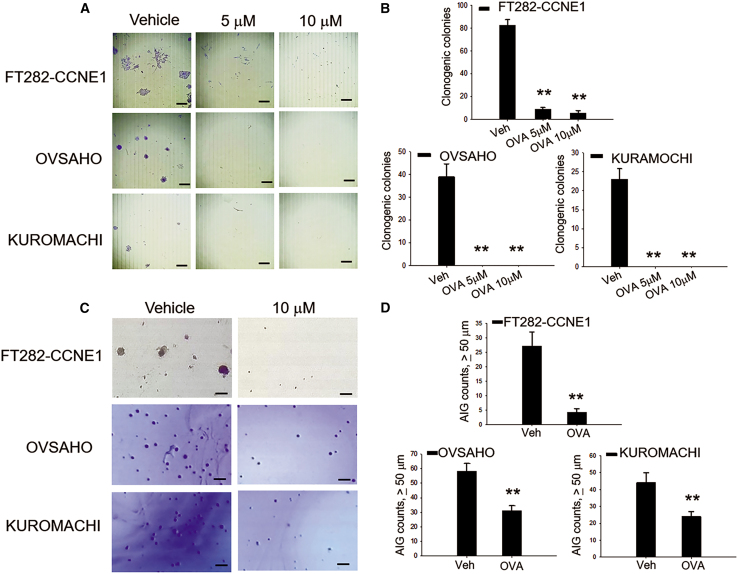
Ovatodiolide significantly suppresses the clonogenicity and anchorage-independent growth of STIC-mimicking cells and HGSC cells (A and B) Representative images and quantification data of cell colonies in STIC-mimicking cells (FT282-CCNE1) and HGSC cells (OVSAHO and KURAMOCHI) after treatment with 5 μM or 10 μM ovatodiolide. Scale bars, 200 μm. (C and D) Representative images and quantification data of AIG of cells after treatment with 10 μM ovatodiolide. Scale bars, 100 μm. ∗∗*p* < 0.01 compared to vehicle medium control.

We further investigated the effect of ovatodiolide on clonogenicity in these STIC-mimicking and HGSC cells. After treatment with either 5 μM or 10 μM, a significant reduction in colony numbers and sizes was observed (Figures 2A and 2B). The two HGSC cells were more sensitive to the treatment than the STIC-mimicking cells. Anchorage-independent growth (AIG) is a hallmark of transformed cells and correlates with tumorigenic potential. We tested the ability of ovatodiolide to inhibit AIG in these STIC-mimicking and HGSC cells and found that AIG was significantly suppressed in all three cell lines (Figures 2C and 2D). Indeed, the HGSC cells (OVSAHO and KURAMOCHI) grew more AIG colonies than the STIC-mimicking cell FT282-CCNE1. The HGSC cells were also more resistant to 10 μM ovatodiolide treatment than the STIC-mimicking cells (Figure 2D).

### Ovatodiolide suppresses OVSAHO-induced orthotopic ovarian HGSC

**Figure 3:**
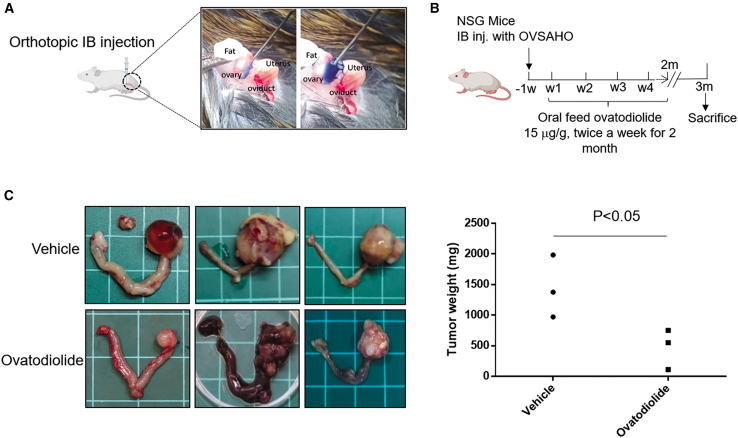
Ovatodiolide suppresses *in vivo* HGSC tumor growth (A) Illustration of the orthotopic xenograft model by intra bursa (IB) (purple dye) injection in NSG mice. (B) Schematic representation of the experimental workflow: OVSAHO cancer cells (2 × 10^6^/10 μL saline) were injected into NSG mice, followed by oral administration of ovatodiolide. (C) Representative images of ovarian tumors and quantitative analysis of tumor weight in mice with or without ovatodiolide treatments. The grid is 1 cm square. ∗*p* < 0.05 compared to vehicle corn oil feed.

In an orthotopic xenograft model, OVSAHO cells (2 × 10^6^ cells in 10 μL saline) were injected into the ovarian bursa of NSG mice (Figure 3A). Following injection, ovatodiolide (or vehicle, 100 μL corn oil) was administered orally twice a week at a dose of 15 μg/g of body weight (Figure 3B). As shown in Figure 3C, the ovatodiolide treatment group exhibited significantly lower tumor growth than the vehicle group. These results demonstrate the tumor-suppressive effect of ovatodiolide *in vivo*.

### RNA-seq identifies 24 differentially expressed genes after ovatodiolide treatment

**Figure 4:**
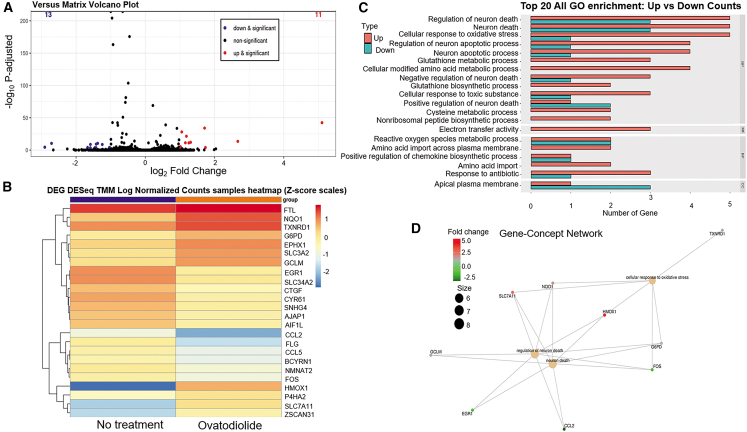
RNA-seq analysis reveals significant differential expression of 24 genes in OVSAHO cells following ovatodiolide treatment and their associated Gene Ontology (GO) classifications (A) Volcano plot showing 11 upregulated (red) and 13 downregulated (blue) genes in OVSAHO cells after ovatodiolide treatment. Genes were considered significant with log_2_ fold change >1 or <−1 with adjusted *p* value (p.adjust) < 0.005. (B) Heatmap of differentially expressed genes. The horizontal axis represents the various sample groups, while the vertical axis corresponds to the genes within the samples. Red indicates high expression levels, whereas blue indicates low expression levels. (C) Bar chart of the top 20 GO enrichment categories, showing positive (orange) and negative (green) regulations. The vertical axis represents GO categories, and the horizontal axis indicates the number of genes. (D) Cnetplot visualization from GO analysis highlights the involvement of nine related genes in the top three biological functions.

To further investigate the mechanism underlying HGSC cell death induced by ovatodiolide, we performed RNA-seq analysis on OVSAHO cells treated with 5 μM ovatodiolide and untreated controls. As shown in Figures 4A and 4B, the treatment resulted in the differential expression of 24 genes, with 11 genes showing upregulation and 13 genes showing downregulation. Table 1 lists the detailed information about these DEGs, ranked by the fold of change. Further Gene Ontology (GO) analysis revealed that ovatodiolide primarily targets processes related to neuron death, oxidative stress, and glutathione metabolism (Figure 4C). Figure 4D features a Centplot visualization, illustrating the involvement of nine genes in the top three processes, including neuron death regulation and oxidative stress responses, showcasing their interconnected roles in specific biological functions (https://figshare.com/s/a86b31fda62f88bc44ff). Notably, HMOX1, the most significantly upregulated DEG, plays a key role in neuron death regulation and oxidative stress responses.

**Table 1:** The significant differential expression genes in OVSAHO cells treated with ovatodiolide

ensembl_gene_id	Symbol	Entrezgene	Description	BaseMean	Log2foldchange	*P* value	padj	OVSA	OVSA_ova
ENSG00000100292	HMOX1	3162	heme oxygenase 1	97.41728075	5.103986284	3.65E−46	4.12E−43	5.5051	189.3295
ENSG00000151012	SLC7A11	23657	solute carrier family 7 member 11	58.75413168	2.671875272	6.12E−17	3.07E−14	15.93858	101.5697
ENSG00000235109	ZSCAN31	64288	zinc finger and SCAN domain containing 31	39.86532985	1.736621021	6.21E−07	0.000112	18.40276	61.3279
ENSG00000181019	NQO1	1728	NAD(P)H quinone dehydrogenase 1	270.4282109	1.712330174	1.07E−37	9.07E−35	126.46	414.3964
ENSG00000168003	SLC3A2	6520	solute carrier family 3 member 2	164.5024558	1.309457512	2.03E−15	9.14E−13	94.58286	234.422
ENSG00000023909	GCLM	2730	glutamate-cysteine ligase modifier subunit	165.3284756	1.256330437	1.71E−14	7.03E−12	97.57135	233.0856
ENSG00000198431	TXNRD1	7296	thioredoxin reductase 1	331.4255278	1.187140777	5.67E−25	3.49E−22	202.2731	460.5779
ENSG00000143819	EPHX1	2052	epoxide hydrolase 1	194.1755093	1.157245506	1.17E−14	4.97E−12	120.2209	268.1301
ENSG00000087086	FTL	2512	ferritin, light polypeptide	531.4243887	1.052302074	1.15E−31	8.67E−29	345.7727	717.0761
ENSG00000160211	G6PD	2539	glucose-6-phosphate dehydrogenase	128.3962676	1.016760904	2.48E−08	6.33E−06	84.93583	171.8567
ENSG00000072682	P4HA2	8974	prolyl 4-hydroxylase subunit alpha 2	71.36315605	1.010546448	3.59E−05	0.004457	47.34386	95.38245
ENSG00000126878	AIF1L	83543	allograft inflammatory factor 1 like	100.695753	−1.029186378	1.43E−06	0.000236	135.1633	66.22819
ENSG00000281398	SNHG4	NA	small nucleolar RNA host gene 4	108.7703465	−1.029620065	5.36E−07	0.000102	146.0162	71.52446
ENSG00000196581	AJAP1	55966	adherens junctions associated protein 1	101.0409423	−1.041350968	1.04E−06	0.00018	136.0022	66.07969
ENSG00000157765	SLC34A2	10568	solute carrier family 34 member 2	143.0537765	−1.242724911	6.68E−12	2.1E−09	201.1197	84.98789
ENSG00000120738	EGR1	1958	early growth response 1	139.3953295	−1.376658578	9.83E−14	3.7E−11	201.277	77.51371
ENSG00000142871	CYR61	3491	cysteine rich angiogenic inducer 61	118.7522012	−1.41901122	1.66E−12	5.61E−10	172.8601	64.64426
ENSG00000157064	NMNAT2	23057	nicotinamide nucleotide adenylyltransferase 2	58.02474436	−1.425659998	7.19E−07	0.000128	84.56883	31.48066
ENSG00000170345	FOS	2353	fos proto-oncogene, AP-1 transcription factor subunit	50.89224663	−1.460713578	2.1E−06	0.00033	74.65964	27.12485
ENSG00000118523	CTGF	1490	connective tissue growth factor	93.07392604	−1.585331997	5.31E−12	1.75E−09	139.6198	46.52802
ENSG00000236824	BCYRN1	618	brain cytoplasmic RNA 1	43.60556184	−1.587054736	2.3E−06	0.000354	65.43205	21.77908
ENSG00000112782	CLIC5	53405	chloride intracellular channel 5	45.80320449	−1.667974731	4.27E−07	8.39E−05	69.67884	21.92757
ENSG00000143631	FLG	2312	filaggrin	46.8186541	−2.708590105	1.06E−13	3.89E−11	81.21334	12.42397
ENSG00000108691	CCL2	6347	C-C motif chemokine ligand 2	21.21698463	−2.904581012	1.67E−07	3.64E−05	37.43468	4.999288

EntrezGene: NCBI gene name.

BaseMean: total sample normalized expression mean.

log2FoldChange:log_2_(Mean_Trt/Mean_Ctrl).

OVSA:OVSAHO mean of standardized expression levels.

OVSA_ova: Ovatodiolide-treated OVSAHO mean of standardized expression levels.

### KEGG pathway analysis reveals that ovatodiolide induces OVSAHO cell death primarily through ferroptosis

**Figure 5:**
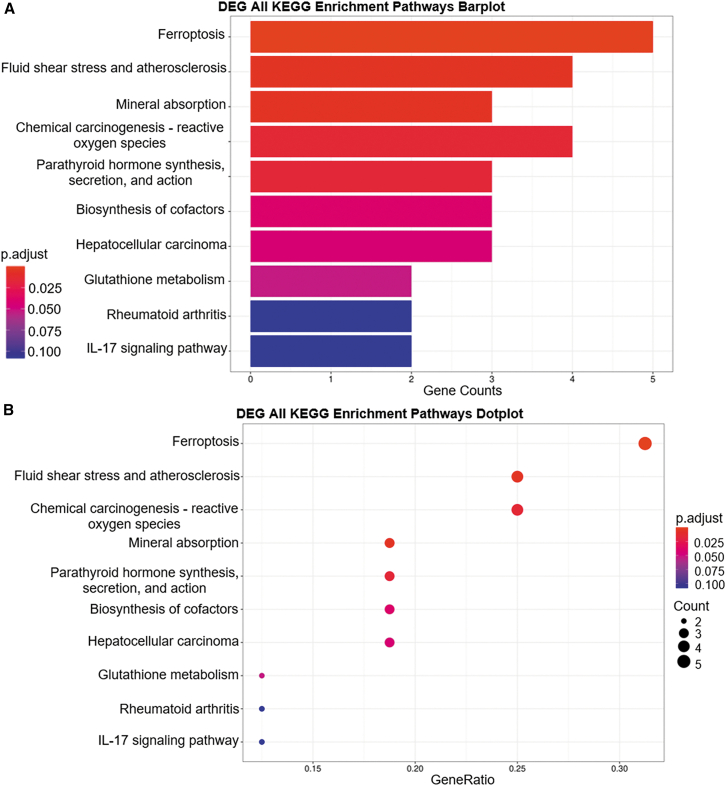
KEGG pathway analysis reveals that ovatodiolide primarily induces OVSAHO cell death through the ferroptosis pathway (A) Bar plot of metabolic pathway enrichment showing the top 10 significant pathways ranked by p.adjust. The vertical axis lists the pathway names, the horizontal axis indicates the number of differentially expressed genes within each pathway, and the color gradient represents the p.adjust values. (B) Dot plot of KEGG pathway enrichment highlighting the top 10 significant pathways ranked by p.adjust. The horizontal axis shows the GeneRatio, while the vertical axis lists the pathways. The color gradient represents p.adjust values, and dot size indicates the gene count.

KEGG pathway analysis further identified specific biological pathways of these differentially expressed genes (DEGs). Figure 5A displays the top 10 significant pathways, ranked by adjusted *p* values. The most significantly enriched pathway is ferroptosis, (p.adjust = 8.09E-07) which is closely linked to cell death and includes the following genes: heme oxygenase 1 (HMOX1), solute carrier family 7 member 11 (SLC7A11), solute carrier family 3 member 2 (SLC3A2), glutamate-cysteine ligase modifier subunit (GCLM), and ferritin (FTL) (Table 2). Figure 5B presents an alternative visualization using a dot plot. Notably, ferroptosis, located at the top-right corner of the plot, shows the most significant relevance. Table 2 provides detailed statistical information about these top 10 KEGG pathways, including specific gene associations and enrichment metrics. As a result, the analysis strongly suggests that ovatodiolide primarily induces cell death through the ferroptosis pathway, a form of regulated cell death characterized by iron-dependent lipid peroxidation.^15^

**Table 2:** Top 10 KEGG metabolic pathway classifications of differentially expressed genes

ID	Description	GeneRatio.Item	GeneRatio.List	BgRatio.Item	BgRatio.List	*P* value	p.adjust	q value	Symbol	Count
hsa04216	ferroptosis	5	16	41	8191	1.02E−08	8.09E−07	6.69E−07	HMOX1/FTL/SLC7A11/SLC3A2/GCLM	5
hsa05418	fluid shear stress and atherosclerosis	4	16	139	8191	0.000123	0.004873	0.00402535	HMOX1/NQO1/CCL2/FOS	4
hsa04978	mineral absorption	3	16	60	8191	0.000196	0.005149	0.00425408	HMOX1/FTL/SLC34A2	3
hsa05208	chemical carcinogenesis—reactive oxygen species	4	16	223	8191	0.000752	0.014855	0.01227168	HMOX1/NQO1/EPHX1/FOS	4
hsa04928	parathyroid hormone synthesis, secretion and action	3	16	106	8191	0.001044	0.016488	0.01362133	EGR1/SLC34A2/FOS	3
hsa01240	biosynthesis of cofactors	3	16	153	8191	0.002993	0.039411	0.03255771	NQO1/GCLM/NMNAT2	3
hsa05225	hepatocellular carcinoma	3	16	168	8191	0.003899	0.044006	0.03635373	HMOX1/NQO1/TXNRD1	3
hsa00480	glutathione metabolism	2	16	57	8191	0.005363	0.052961	0.04375225	GCLM/G6PD	2
hsa05323	rheumatoid arthritis	2	16	93	8191	0.0138	0.108276	0.08944866	CCL2/FOS	2
hsa04657	IL-17 signaling pathway	2	16	94	8191	0.014084	0.108276	0.08944866	CCL2/FOS	2

GeneRatio Item: number of genes annotated to specific gene groups.

GeneRatio List: number of genes annotated to the overall gene pool.

BgRatio Item: number of genes in a specific gene group.

BgRatio List: total number of genes in the overall gene pool.

qvalue: FDR (false discovery rate) calculated q value.

### Validation of ferroptosis-related changes after ovatodiolide treatment

**Figure 6:**
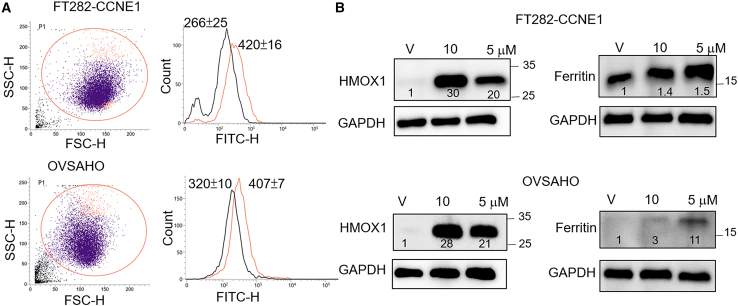
Validation of ROS and iron-metabolism-related marker involvement in ferroptosis in cells following ovatodiolide treatment (A) Flow cytometry analysis of intracellular ROS levels (mean fluorescence) in cells stained with 2 μM DCFDA after treatment with 5 μM ovatodiolide for 15 h (pink). (B) Western blot analysis of HMOX1 and Ferritin expression in cells treated with ovatodiolide for 15 h.

Ferroptosis is mediated by three key pathways: iron metabolism, redox regulation, and lipid metabolism.^16^ Dysregulation of oxidative-reductive systems, iron metabolism, and the peroxidation of polyunsaturated fatty acids (PUFAs) can induce ferroptosis.^15^ We first examined intracellular reactive oxygen species (ROS) levels post-treatment with 5 μM ovatodiolide for 15 h. We observed a significant increase in ROS levels, with a 58% rise in FT282-CCNE1 cells and a 27% increase in OVSAHO cells (Figure 6A). Next, we validated the upregulation of HMOX1, which ranked first in the differential gene expression list, along with ferritin, which was also upregulated in the RNA-seq results (Table 1). HMOX1 catalyzes the degradation of heme, releasing free iron that is thought to promote Fenton reactions, leading to a surge in intracellular ROS and triggering ferroptosis.^17^^,^^18^^,^^19^ Meanwhile, studies have reported that HMOX1 can regulate ferritin to sequester iron, thereby mitigating its pro-oxidant effects.^16^^,^^20^ After treatment, we measured HMOX1 and ferritin protein levels in FT282-CCNE1 and OVSAHO cells. HMOX1 levels increased 30- and 20-fold in FT282-CCNE1 and 28- and 21-fold in OVSAHO cells, whereas ferritin levels rose 1.4- and 1.5-fold in FT282-CCNE1 and 3- and 11-fold in OVSAHO cells, following 10 μM and 5 μM ovatodiolide treatments, respectively (Figure 6B). These results support the role of ovatodiolide in inducing ferroptosis through the modulation of oxidative stress and HMOX1 expression.

## Discussion

In recent years, targeted therapies, particularly poly(ADP-ribose) polymerase (PARP) inhibitors, have emerged as effective treatments for high-grade serous carcinoma (HGSC) of the ovary, especially in tumors with defective homologous recombination repair or BRCA1/2 mutations.^21^^,^^22^ However, challenges such as high costs, side effects, and the development of drug resistance continue to pose significant issues. There is a pressing need to develop additional cost-effective and efficient treatments to combat this devastating gynecological cancer.

Ovatodiolide, a natural compound extracted from the traditional medicinal herb, has been shown in previous studies to exhibit diverse anti-cancer properties.^9^^,^^10^^,^^11^^,^^12^^,^^13^ This study is the first to reveal its cytotoxic effects on HGSC and its precursor STIC cells. Our data demonstrated that ovatodiolide exhibits dose-dependent cytotoxicity against both STIC-mimicking and HGSC cells, outperforming cisplatin at the same concentration. In addition to the cytotoxicity activity, a concentration of 5 μM of ovatodiolide nearly completely abolished the colony formation capabilities of both cell types, consistent with its stemness-suppressing activity observed in other cancer cells.^9^^,^^11^^,^^13^ Moreover, a dose of 10 μM *in vitro* and 15 μg/g body weight *in vivo* effectively reduced the anchorage independent growth and tumorigenicity of OVSAHO cells. The *in vivo* dose used was well below the lowest oral toxicity dose of 1,000 μg/g body weight in rats, with no significant adverse effects noted at doses of 10–50 μg/g.^14^ Nevertheless, the evaluation of ovatodiolide’s potential cytotoxicity in non-cancerous cells, such as primary human fimbrial epithelial cells or fibroblasts, remains limited in this study. Future studies are warranted to assess the compound’s selectivity and safety profile in normal tissues, which will be essential for its advancement as a clinically relevant anticancer agent.

RNA-seq analysis identified 24 DEGs in ovatodiolide-treated OVSAHO cells, with 11 genes upregulated and 13 downregulated. GO analysis revealed associations with neuronal death, oxidative stress, and glutathione metabolism. Additionally, KEGG pathway analysis indicated that ferroptosis is the primary mechanism of cell death.

Ferroptosis is a distinct, non-apoptotic form of regulated cell death driven by iron-dependent lipid peroxidation. Studies have shown that therapy-resistant cancer cells, particularly dedifferentiated or stem-like cells, are highly susceptible to ferroptosis, positioning it as a promising therapeutic strategy for refractory cancers.^23^^,^^24^ Our research demonstrated that ovatodiolide treatment induced death in HGSC and its precursor cells, which exhibit high expression of HMOX1. This key enzyme in heme metabolism promotes Fe^2+^ accumulation, subsequently triggering the Fenton reaction and ferroptosis.^16^^,^^18^^,^^19^

Our previous research has shown that ovulatory ROS plays a causal role in the transformation of the fallopian tube fimbrial epithelium, which is exposed to ROS and carcinogens present in the follicular fluid.^25^^,^^26^^,^^27^ Ovulation is characterized by acute inflammation and requires a high level of ROS in the follicular fluid.^28^ Moreover, the fallopian tube fimbria is constantly immersed in peritoneal fluid that contains high levels of iron, with concentrations approximately 60 mg/mL—60 times higher than the serum level of about 1 mg/mL.^29^^,^^30^ Consequently, the fimbrial epithelium exists in an environment conducive to the Fenton reaction. It is therefore assumed that a robust antioxidant machinery is in place to counteract oxidative stress, maintain cell viability, and facilitate malignant transformation. Treatment with ovatodiolide may disrupt this antioxidant system, exposing the cells to excessive ROS and promoting ferroptosis. However, this study did not assess key antioxidant factors such as glutathione (GSH) and glutathione peroxidase 4 (GPX4), limiting our ability to further elucidate the mechanistic link between HMOX1 upregulation and the initiation of ferroptosis. Future investigations examining GSH-related antioxidant responses will be valuable for deepening our understanding of the anticancer mechanism of ovatodiolide.

### Conclusions

In conclusion, this study identified ovatodiolide, a compound derived from Anisomeles indica, as a promising therapeutic agent for HGSC and its precursor lesion STIC. The compound demonstrates anti-cancer effects primarily by inducing ferroptosis through the overexpression of HMOX1. This mechanism presents an intriguing area for future research.

## Materials and methods

### Cells

Two immortalized human fallopian tube epithelial cells were utilized in this study. FT282-CCNE1 were immortalized by hTERT plus a dominant-negative *TP53-R175H* mutation, with additional transduction with *CCNE1*. The cells were kindly donated by Dr. Drapkin R.^31^ Cells were maintained in MCDB105 and M199 media (Sigma) supplemented with 10% fetal bovine serum (FBS) and P/S. Two human high-grade serous carcinoma cell lines, OVSAHO and KURAMOCHI, were purchased from JCRB Cell Bank and were cultured in RPMI-1640 medium with 10% FBS, 100 IU/mL of penicillin, and 100 μg/mL of streptomycin.

### Reagents

Ovatodiolide was extracted from Anisomeles indica using standard extraction techniques, and its macrocyclic diterpenoid structure was confirmed by nuclear magnetic resonance (NMR) spectroscopy, as described in previously published protocols.^32^ The purity of the extracted compound was analyzed using high-performance liquid chromatography (HPLC) and determined to be 98%.^33^ The purified ovatodiolide was then dissolved in dimethyl sulfoxide (DMSO) (Cat. No. D2650, SIGMA) to prepare a 100 mM stock solution, which was stored at −20°C until further use. Cisplatin (CDDP) was purchased from Fresenius Kabi Taiwan (Taipei) under the product name KEMOPLAT, with a concentration of 50 mg/100 mL per vial, and was stored at −4°C, protected from light.

### Western blot analysis

Protein concentrations of RIPA-lysed cell lysates were determined using the Protein Assay Dye Reagent (BIO-RAD, 500-0006). Samples were mixed with an equal volume of 2× Laemmli sample buffer, heated at 95°C for 5 min, and then cooled on ice for 15 min. A total of 30 μg of crude protein extract was separated by SDS-PAGE and transferred onto a nitrocellulose membrane. The membrane was probed with primary antibodies against HO-1/HMOX1 (10701-1-AP, proteintech), Ferritin light chain (A11241, Abclonal), and GAPDH (#2118, Cell Signaling). After washing with Tris-buffered saline containing 0.05% Tween 20 (TBST), the membrane was incubated with horseradish peroxidase (HRP)-conjugated secondary antibodies and visualized using the ECL Western Blot Detection Reagent (GE Healthcare, RPN2209).

### Clonogenicity assay

The clonogenicity assay was performed as previously described to assess the ability of single cells to form colonies in a two-dimensional (2D) adherent culture.^34^ A total of 500 cells were seeded into each well of a 6-well plate and incubated in a standard culture medium with 10% FBS for 4 h to allow cell attachment. The medium was then replaced with serum-free medium for subsequent experiments. The test reagents were added to the wells, and the cells were incubated for 7 days. After the incubation period, the cell colonies were fixed with 4% paraformaldehyde and stained with 0.5% crystal violet. Colonies were then counted and compared between groups under a microscope.

### Anchorage-independent growth assay

The anchorage-independent growth (AIG) assay was performed to evaluate the formation of transformed cell colonies. A 6-well plate was prepared with a bottom layer of 0.8% agarose (0.5 mL per well); 0.5mL of culture medium containing 0.4% agarose and 2,000 suspended cells was then added to the upper layer. The culture medium was replenished every 2 days, and the test reagents were added once daily during the first 2 days. After 2 weeks of incubation, the spheroid colonies were fixed with 4% paraformaldehyde and stained with 0.5% crystal violet. Colonies larger than 50 μm were counted under a microscope.

### Cell viability assay

Cell viability was assessed using the XTT assay (20-300-1000A, BioLegend) as previously described.^25^ Briefly, 7,000 cells were seeded into each well of a 96-well plate. After overnight incubation, the cells were treated with the test reagents and cultured for 24 h. Following treatment, the XTT reagent was added according to the manufacturer’s instructions, and the absorbance was measured at 450 nm using a microplate reader to evaluate cell viability.

### Flow cytometry analysis of cellular ROS

3 × 10^5^ cells were seeded per well in a 6-well plate and allowed to adhere overnight. The cells were treated with 5 μM ovatodiolide for 15 h under standard culture conditions. After treatment, the cells were detached and stained with 2 μM DCFDA (2′,7′-dichlorofluorescin diacetate) for 30 min at 37°C to detect intracellular ROS. Cells were then washed thoroughly with PBS to remove excess dye, resuspended in 700 μL of PBS, and transferred to a flow cytometry tube. The stained cells were analyzed using a flow cytometer with a 488 nm excitation laser and a 530 nm emission detection channel, and the mean fluorescence intensity (MFI) was quantified to assess and compare ROS levels between treated and control groups.

### Mouse xenograft tumorigenic analysis

For the cell xenograft transplantation, 2 × 10^6^ OVSAHO cells in 10 μL of medium were injected into the ovarian bursa of immunocompromised NOD/Shi-scid/IL-2Rγnull (NSG) mice. One week after transplantation, the mice were orally administered ovatodiolide, dissolved in corn oil, at a dose of 15 μg/g body weight, twice a week, for two months. At the end of the third month, the mice were sacrificed, and tumor weights were measured and compared between the ovatodiolide-treated and untreated groups. All experimental procedures were performed under the guidelines of the Animal Care and Use Committee of Tzu Chi General Hospital (Approval ID: 111-51).

### RNA-seq analysis

To understand the mechanism by which ovatodiolide induces toxicity in HGSC cells, OVSAHO cells treated with 5 μM ovatodiolide for 15 h were selected for RNA-seq analysis (BIOTOOLS) to examine gene expression levels, comparing them to a control group treated with vehicle medium. The analysis first used edgeR for trimmed mean of M-values (TMM) normalization of the data, followed by DEGseq for differential gene analysis.

Differential gene clustering analysis was then conducted to identify genes with similar expression patterns of all DEGs, as genes grouped may share similar functions or participate in the same biological processes. GO analysis was applied to classify these DEGs into three main categories: molecular function (MF), biological process (BP), and cellular component (CC). Finally, KEGG pathway analysis was performed to summarize the primary biological signaling pathways associated with these DEGs, providing insights into the molecular mechanisms underlying ovatodiolide-induced toxicity.

### Statistical analysis

Statistical analysis was performed using GraphPad Prism (ver. 5.0c) (GraphPad Software, San Diego, CA, USA), Excel, or SPSS 19.0. Differences between groups were examined using unpaired Student’s t tests and one-way analysis of variance. A *p* value less than 0.05 is considered statistically significant in the comparison.

## Data availability

Data will be made available on request.

## Acknowledgments

This study was supported by grants from the 10.13039/501100004737National Health Research Institutes (NHRI-EX112-11216BI to T.Y.C.), National Science and Technology Council, (NSTC 112-2314-B-303-002-MY3 to T.Y.C., NSTC 112-2314-B-303 -011-MY3 to H.S.H., and NSTC 112-2811-B-303-007 to A.A.K.), Taiwan, ROC; and Tzu Chi General Hospital (TCRD112-044 to H.S.H.), Hualien, Taiwan. The authors acknowledge the core facilities provided by the Advanced Instrumentation Center of the Department of Medicine Research, Hualien Tzu Chi Hospital, Buddhist Tzu Chi Medical Foundation, Hualien, Taiwan. Some figures were created using BioRender and ChemSpider.

## Author contributions

Conceptualization, H.S.H., Y.M.T., and T.Y.C.; methodology, A.A.K., Y.M.T., and H.S.H.; investigation, Y.M.T. and H.S.H.; writing—original draft, H.S.H.; writing—review & editing, H.S.H., Y.M.T., and T.Y.C.; funding acquisition & resources, Y.M.T., T.Y.C., and H.S.H.; supervision, T.Y.C.

## Declaration of interests

The authors declare that they have no known competing financial interests.
