# Ovatodiolide triggers ferroptosis in high-grade serous ovarian cancer through HMOX1 upregulation

**DOI:** 10.1016/j.omton.2025.201059

**Published:** 2025-09-20

**Authors:** Yew-Min Tzeng, Aye Aye Khine, Hsuan-Shun Huang, Tang-Yuan Chu

## Main text

(Molecular Therapy: Oncology *33*, 201023; September 2025)

In the originally published version of this paper, an animal experiment approval ID was omitted. The corresponding ID number (113-08) has been added to the mouse xenograft tumorigenic analysis section and now appears in the corrected article online.

The authors regret this error.

